# The efficacy of probiotics for monosodium glutamate-induced obesity: dietology concerns and opportunities for prevention

**DOI:** 10.1186/1878-5085-5-2

**Published:** 2014-01-13

**Authors:** Oleksandr A Savcheniuk, Oleksandr V Virchenko, Tetyana M Falalyeyeva, Tetyana V Beregova, Lidia P Babenko, Liudmyla M Lazarenko, Olga M Demchenko, Rostyslav V Bubnov, Mykola Ya Spivak

**Affiliations:** 1 Taras Shevchenko National University of Kyiv, Volodymyrska Str., 64/13, Kyiv 01601, Ukraine; 2 Zabolotny Institute of Microbiology and Virology, National Academy of Sciences of Ukraine, Zabolotny Str., 154, Kyiv 03680, Ukraine; 3 LCL ‘DIAPROF’, Svitlycky Str., 35, Kyiv 04123, Ukraine; 4 Clinical Hospital ‘Pheophania’ of State Affairs Department, Zabolotny str., 21, Kyiv 03680, Ukraine

**Keywords:** Predictive, preventive, personalized medicine, obesity, probiotics, animal model, monosodium glutamate, food additives, personalized dietology

## Abstract

**Introduction:**

Obesity becomes endemic today. Monosodium glutamate was proved as obesogenic food additive. Probiotics are discussed to impact on obesity development.

**Aims and objectives:**

The aim was to study the effects of probiotics on the development of monosodium glutamate (MSG)-induced obesity in rats.

**Material and methods:**

We included 45 Wistar male rats and divided into three groups (*n* = 15). Newborn rats of group 1 (control) received subcutaneously 8 μl/g saline. Group 2 received 3 to 4 mg/g MSG subcutaneously on the second, fourth, sixth, eighth and tenth day of life. Within 4 months after birth, rats were on a standard diet. Group 3 received an aqueous solution of probiotics mixture (2:1:1 *Lactobacillus casei* IMVB-7280, *Bifidobacterium animalis* VKL, *B. animalis* VKB) at the dose of 5 × 10^9^ CFU/kg (50 mg/kg) intragastrically. Administration of probiotics was started at the age of 4 weeks just after weaning and continued for 3 months during 2-week courses. Group 2 received intragastrically 2.5 ml/kg water. Organometric and biochemical parameters in all groups of rats were analyzed over 4 months. The concentration of adiponectin was determined in serum, and leptin - in adipose tissue.

**Results:**

Administration of MSG led to the development of obesity in rats; body weight had increased by 7.9% vs controls (*p* < 0.05); body length had increased by 5.4% (*p* < 0.05). Body mass index and Lee index and visceral fat mass had increased (*p* < 0.001). Under the neonatal injection of MSG, the concentration of total cholesterol, triglycerides, VLDL cholesterol and LDL cholesterol significantly increased (*p* < 0.001), in comparison with controls. Adipose-derived hormones changed in MSG obesity rats: adiponectin decreased by 58.8% (*p* < 0.01), and leptin concentration in adipose tissue had increased by 74.7% (*p* < 0.01). The probiotic therapy of rats from group 3 prevented obesity development. Parameters of rats treated with probiotic mixture did not differ from that in the control.

**Conclusions:**

The introduction of MSG to newborn rats caused the obesity in adulthood. Periodic administration of probiotic mixture to rat injected with MSG neonatally resulted in recovery of lipid metabolism and prevention of the obesity development.

## Overview

### Predictive, preventive and personalized anti-obesity concerns

Metabolism is an essential process for the maintenance of life and homeostasis of the organism. Diseases associated with metabolic disorders such as hyperlipidemia, diabetes and obesity have become extremely common [1,2]. Today, obesity becomes endemic; about 1.7 billion people on the planet are overweight. World Health Organization (WHO) has declared obesity a global epidemic and took it under control [1]. Overweight and obesity cause the leading severe diseases, namely, diabetes mellitus type 2, cardiovascular diseases (CVD) and breast cancer [3]. The permanently growing cohort of patients with obesity-related diseases, as diabetes, requires the urgent change paradigm from interventional measures to predictive, preventive and personalized medicine (PPPM) [4,5].

Metabolic disturbances in obesity causes a number of diseases, namely, cardiovascular diseases (hypertension, atherosclerosis, coronary heart disease), stroke, insulin-dependent diabetes, premature death, diseases of musculoskeletal system (osteochondrosis and metabolic-dystrophic arthritis), hepatobiliary disease (gallbladder dyskinesia, chronic cholecystitis, cholelithiasis) and number of tumor sites, including lung cancer, breast cancer, uterine cancer and ovarian; in women, there is a violation of ovarian-menstrual cycle dyslipidemia. Obesity reduces life expectancy by 3 to 5 and, sometimes, in severe forms, for 15 years [6,7].

Health and disease of individuals and of populations are the result of three groups of factors: genetics, environment and behavior. Only the latter is mostly dependent by the choices of the single person, but assessment, interventions and tailored changes are possible [8]. Many of the researchers attribute this to a violation of the diet. The nature of nutrition today is a serious concern; a growing consumption of ‘fast food’, accompanied by a decrease in the proportion of the daily diet of vegetables, fruits, milk and dairy products seriously affects the health. The application of food supplement for metabolic syndrome was considered for PPPM in [9].

The recent data, regarding women’s differential responses to lifestyle changes, support another branch of research with a gender nutrition emphasis within predictive, preventive and personalized medicine [10].

Over the past 10 years, morbidity diseases of the gastrointestinal tract in children and adults have increased. In 2011, in Ukraine, 7,089,010 patients were diagnosed with gastroenterological diseases, per 100,000 of the adult population, 18956.3. At the end of 2011, the records were 5,028,034 patients with diseases of the digestive organs, i.e. 70.9% of the total registered. In 2006, there was an increased prevalence of 11.2% [11].

### Monosodium glutamate

Monosodium glutamate (MSG, C_5_H_8_NO_4_Na, E 621) is widely distributed and is naturally occurring in various foods (bouillon cubes, meat tenderizers, canned food, frozen food, potato and snack chips, barbecue sauce, salad dressing, soups, fast food, etc.) [12].

Obesogenic properties of monosodium glutamate were studied for decades [13,14]. Hirata et al. demonstrated that MSG obese rats develop insulin resistance to peripheral glucose uptake [15]. MSG induces hyperinsulinemia in 3-month-old rats; the obesity of MSG animals is a metabolic alteration characterized by an enhanced adipocyte capacity to transport glucose and to synthetize lipids resulting in increased insulin sensitivity [16]. It was supposed that the central lesions produced by MSG treatment disrupt the regulation of the hypothalamic-pituitary-adrenal axis as the hyperfunctional state of adrenals revealed signs of MSG-treated rats [17].

In Ukraine, monosodium glutamate became a legal food additive (flavour boosters) *only in 2000* after the Resolution of Cabinet of Ministers of Ukraine no. 342 on February 17, 2000 ‘Amendments to the list of food additives authorized for use in foodstuffs’ [18], which authorized the list of food additives in Ukraine.

Now, it is hard to find industrially produced canned or semi-finished products that do not include MSG. Thus, the permissible limits may be significantly exceeded, which can lead to diseases of the digestive tract.

### Probiotics

Therefore, the search of new non-toxic means of the obesity prevention is the urgent challenge of modern science. Today, the question of the probiotics influence on lipid metabolism and obesity is actively debated in the scientific literature [19-21]. Backhed et al. were the pioneers in the study of the role of colon microflora in the metabolism regulation [22]. Their findings were the impetus for the research in this field. Further studies have shown the alteration of intestinal microbiota composition in overweight people. Thus, intestine microbiocenosis can be considered the environmental factor that modulates the development of obesity.

It was demonstrated that prolonged exposure to a high fat diet significantly changed the composition of the colon microflora in mice having reduced the level of *Bifidobacterium* and *Lactobacillus* that are known to produce many positive physiological effects, e.g. improving the barrier function of the intestinal mucosa and having increased levels of *Firmicutes* and *Proteobacteria*, which produce a lot of toxic substances [23,24].

It was found that the oligofructose prebiotic which is a supplement to a high fat diet resulted in the recovery of the normal composition of bifidoflora, hence elimination endotoxemia and reduction of the obesity development. These data suggest that bifidoflora may reduce intestinal permeability and the level of circulating endotoxin. In addition, the growth of bifidobacteria improved the sensitivity to glucose and decreased the body weight gain and production of pro-inflammatory mediators [25-27].

Recently, the beneficial effects of probiotic bacteria on the obesity development was established. For example, the use of *Lactobacillus gasseri* SBT2055 and *Lactobacillus paracasei* SSP paracasei F19 prevented the development of diet-induced obesity [27,28]. The most important cause of obesity is the excessive consumption of fat and easily digestible carbohydrates, but due to accumulated data, scientists believe that the uncontrolled use of food additives such as MSG taste enhancer can also lead to obesity [29].

The aim was to investigate the effect of probiotics on the development of experimental obesity in rats induced by monosodium glutamate.

## Methods

We included 45 Wistar male rats and divided to three groups of 15 animals each. Newborn rats of group 1 (control) were injected with saline with a volume of 8 μl/g subcutaneously on the second, fourth, sixth, eighth and tenth day of life. Newborn rats of groups 2 and 3 were administered with MSG dissolved in saline at the dose 4 mg/g of body weight with a volume of 8 μl/g subcutaneously on the second, fourth, sixth, eighth and tenth day of life [30].

Within 4 months after birth, rats were on a standard diet. Group 3 received an aqueous solution of a mixture of probiotics (2:1:1 *Lactobacillus casei* IMVB-7280, *Bifidobacterium animalis* VKL, *B. animalis* VKB) at a dose of 5 × 10^9^ CFU/kg (50 mg/kg) intragastrically (i.g.). Group 2 received water with a volume of 2.5 ml/kg i.g., respectively.

Group 2 respectively received 2.5 ml/kg of water (intragastrically). Administration of probiotics was started at the age of 4 weeks just after weaning and continued for 3 months, intermittently alternating 2-week course of introduction with 2-week course of break. The changes in body weight in all groups of rats were analyzed for 4 months from birth. Four-month-aged animals were sacrificed. The rats’ blood was collected in tubes, and visceral adipose tissue was removed and weighed. Body length was measured; body mass index (BMI) (the ratio of body weight (g) of rats to the square of the body length (cm^2^)) and Lee obesity index (the ratio of 1/4 of cube root of body weight (g) to body nose-to-anus length (cm)) were calculated.

Blood samples were kept at a temperature of 38°C for at least 30 min and centrifuged for 10 min at 1,000 × *g*, followed by collecting of serum. The concentration of adiponectin was determined in serum, and leptin was measured in adipose tissue by enzyme immunoassay. Adipose tissue was homogenized in the TEC buffer (10 mM TRIS, 1 mM EDTA, 250 mM saccharose, protease inhibitors (2.5 μg/ml leupeptin, 3.5 μg/ml aprotinin, 1 mM phenylmethanesulfonyl fluoride), 1% triton X-100) at the rate of 4 ml buffer per 1 g of tissue. Homogenate was centrifuged for 15 min, and then medium layer (soluble fraction) was harvested. Cholesterol, triglycerides, high-density lipoproteins (HDL cholesterol), low-density lipoproteins (LDL cholesterol), very low-density lipoproteins (VLDL cholesterol), bilirubin, activity of alanine and aspartate aminotransferase in serum were determined by standard biochemical methods. All investigated parameters of all rats were estimated in the same material which is serum, except leptin which was measured in visceral adipose tissue. The concentration of leptin and adiponectin was measured with immunoassay method using ‘BioVendor’ commercial kits (Czech Republic) (Leptin Mouse/Rat Elisa, Adiponectin HMW Mouse/Rat ELISA).

Research was conducted in compliance with the standards of the Convention on Bioethics of the Council of Europe’s ‘Europe Convention for the Protection of Vertebrate Animals’ used for experimental and other scientific purposes’ (1997), the general ethical principles of animal experiments, approved by the First National Congress on Bioethics Ukraine (September 2001) and other international agreements and national legislation in this field. Animals were kept in a vivarium that was accredited in accordance with the ‘standard rules on ordering, equipment and maintenance of experimental biological clinics (vivarium)’. Instruments to be used for research are subject to metrological control.

Statistical analysis of data was carried out by the software package ‘Statistica 8.0’. For the analysis of data distribution type, Shapiro-Wilks criterion was used. As the data were normally distributed, we used Levan criterion for evaluating the equality of variance and Student’s *t* test for independent samples. We calculated mean values (M) and standard deviations (SD). Significant difference was considered at *p* ≤ 0.05.

## Results

Administration of MSG in the neonatal period leads to the development of obesity in 4-month-old rats. Table 1 shows the organopometric parameters in three groups of rats. It was found that after 4 months in animals injected with MSG, body weight was significantly higher in comparison with control animals by 7.9% (*p* < 0.05). This decreased the body length in the group of rats with experimental obesity by 5.4% (*p* < 0.05). Body length in group 2 was decreased by MSG introduction by 5.4% (*p* < 0.05) compared to that of the control. The calculation of body mass index and Lee index suggested the development of obesity in group 2. The significant increase of visceral adipose tissue mass was also observed in animals injected with MSG by 583% (*p* < 0.001) in comparison with that of the control. These data confirm the findings that the introduction of glutamate sodium to newborn rodents induces the development of visceral obesity in adult animals and is a model of obesity in rodents [30].

**Table 1:** Organopometric parameters of glutamate-induced obesity rats and corrected parameters by probiotics

	**Intact rats (*n*** **= 15)**	**MSG-induced obesity**		***р***_**1**_	***р***_**2**_	***р***_**3**_
		**Placebo (*n*** **= 15)**	**Probiotic blends (*n*** **= 15)**			
Weight, g	241.9 ± 25.5	261 ± 17.8	256.7 ± 27.7	0.016	>0.05	>0.05
Body length, cm	21.4 ± 0.9	20.3 ± 1.6	21.5 ± 0.6	0.023	>0.05	0.011
Body mass index	0.53 ± 0.05	0.64 ± 0.08	0.56 ± 0.07	0.002	>0.05	>0.05
Lee index	0.29 ± 0.01	0.32 ± 0.02	0.30 ± 0.01	0.013	>0.05	>0.05
Visceral fat mass, g	2.53 ± 0.78	17.31 ± 5.69	10.65 ± 3.89	<0.001	<0.001	<0.001

Data are presented as the M ± SD. *p*_1_, group 1 vs group 2; *p*_2_, group 1 vs group 3; *p*_3_, group 2 vs group 3. Significant *p* value < 0.05.

While the development of MSG-induced obesity was registered, there were no functional changes in the liver. It was confirmed by determination of bilirubin and albumin concentration and activity of alanine and aspartate aminotransferase in blood serum (Table 2). However, in the blood of animals, injected with MSG, the lipid metabolism changes that are characteristic of the metabolic syndrome are observed. Under the neonatal injection of MSG, the concentration of total cholesterol, triglycerides, VLDL cholesterol and LDL cholesterol significantly increased by 55% (*p* < 0.001), 210% (*p* < 0.001), 210% (*p* < 0.001) and 83% (*p* < 0.001), respectively, compared to those of the controls (Table 3). Also, it was found that MSG introduction influenced HDL cholesterol concentration; it decreased by 33.1% (*p* < 0.001).

**Table 2:** Biochemical indicators of liver in blood serum of glutamate-induced obesity rats and corrected by probiotics

	**Intact rats (*n*** **= 15)**	**MSG-induced obesity**		***р***_**1**_	***р***_**2**_	***р***_**3**_
		**Placebo (*n*** **= 15)**	**Probiotic blends (*n*** **= 15)**			
Alanine aminotransferase, μkat/L	0.228 ± 0.033	0.211 ± 0.031	0.221 ± 0.034	>0.05	>0.05	>0.05
Aspartate transaminase, μkat/L	0.389 ± 0.034	0.377 ± 0.041	0.392 ± 0.044	>0.05	>0.05	>0.05
Total albumin, μmol/L	12.4 ± 2.09	12.7 ± 1.53	12.3 ± 1.98	>0.05	>0.05	>0.05
Indirect bilirubin, μmol/L	7.9 ± 1.7	8.1 ± 1.06	7.7 ± 1.4	>0.05	>0.05	>0.05
Direct bilirubin, mmol/L	4.4 ± 0.91	4.6 ± 0.91	4.6 ± 0.97	>0.05	>0.05	>0.05

Data are presented as the M ± SD. *p*_1_, group 1 vs group 2; *p*_2_, group 1 vs group 3; *p*_3_, group 2 vs group 3. Significant *p* value < 0.05.

**Table 3:** Biochemical parameters of lipid metabolism in serum of glutamate-induced obesity rats and corrected by probiotics

	**Intact rats (*n*** **= 15)**	**MSG-induced obesity**		***р***_**1**_	***р***_**2**_	***р***_**3**_
		**Placebo (*n*** **= 15)**	**Probiotic blends (*n*** **= 15)**			
Triglycerides, mmol/L	1.15 ± 0.27	3.53 ± 0.57	2.91 ± 0.72	<0.001	<0.05	>0.05
Total cholesterol, mmol/L	4.53 ± 0.34	7.04 ± 0.26	4.72 ± 0.37	<0.001	>005	<0.001
Very low-density lipoproteins, mmol/L	0.51 ± 0.12	1.58 ± 0.26	1.07 ± 0.41	<0.001	<0.05	<0.05
HDL cholesterol, mmol/L	1.63 ± 0.14	1.09 ± 0.19	1.37 ± 0.11	<0.001	<0.05	<0.05
LDL cholesterol, mmol/L	2.37 ± 0.22	4.35 ± 0.29	3.02 ± 0.49	<0.001	<0.05	<0.05

Data are presented as the M ± SD. *p*_1_, group 1 vs group 2; *p*_2_, group 1 vs group 3; *p*_3_, group 2 vs group 3. Significant *p* value < 0.05.

Taking into account the literature data that adipose tissue is an active secretory organ and can model the development of obesity, we have investigated the contents of adipose-derived hormones in rats of all groups. Analysis of the secretory function of adipose tissue showed a change in the concentration of adipose-derived hormones in rats with experimental obesity caused by MSG. Thus, the level of adiponectin in the serum of rats with monosodium glutamate-induced obesity decreased by 2.43 times (*p* < 0.01).

These results are confirmed by the literature data, which shows the low levels of adiponectin in humans with obesity and insulin resistance. A study on rhesus monkeys, which simulated obesity and type 2 diabetes, confirmed this statement and showed that adiponectin levels decreased in parallel with the progression of data pathological conditions [31,32]. Scherer Lab set a line of transgenic mice with a threefold increase in adiponectin levels in serum [33]. For this model, hyperadiponectinemia characteristically increased the sensitivity of peripheral tissues to insulin by improving carbohydrate and lipid metabolism associated with increased activation of AMPK in the liver and the expression of peroxisome proliferator-activated receptor-gamma (PPARγ) in visceral adipose tissue. These animals are resistant to the development of insulin resistance induced by intake of a high fat diet [34].

Treatment of animals with recombinant adiponectin with obesity leads to decreased hyperglycemia and free fatty acids (FFA) in plasma and improves insulin sensitivity [35]. Activation of PPARγ *in vivo* leads to increased adiponectin levels [36]. In mice without adiponectin, hepatic insulin resistance was observed in parallel with a decrease in therapeutic response to agonists of PPARγ, indicating that adiponectin is an important factor enhancing PPARγ-mediated improvement in insulin sensitivity [37].

**Figure 1:**
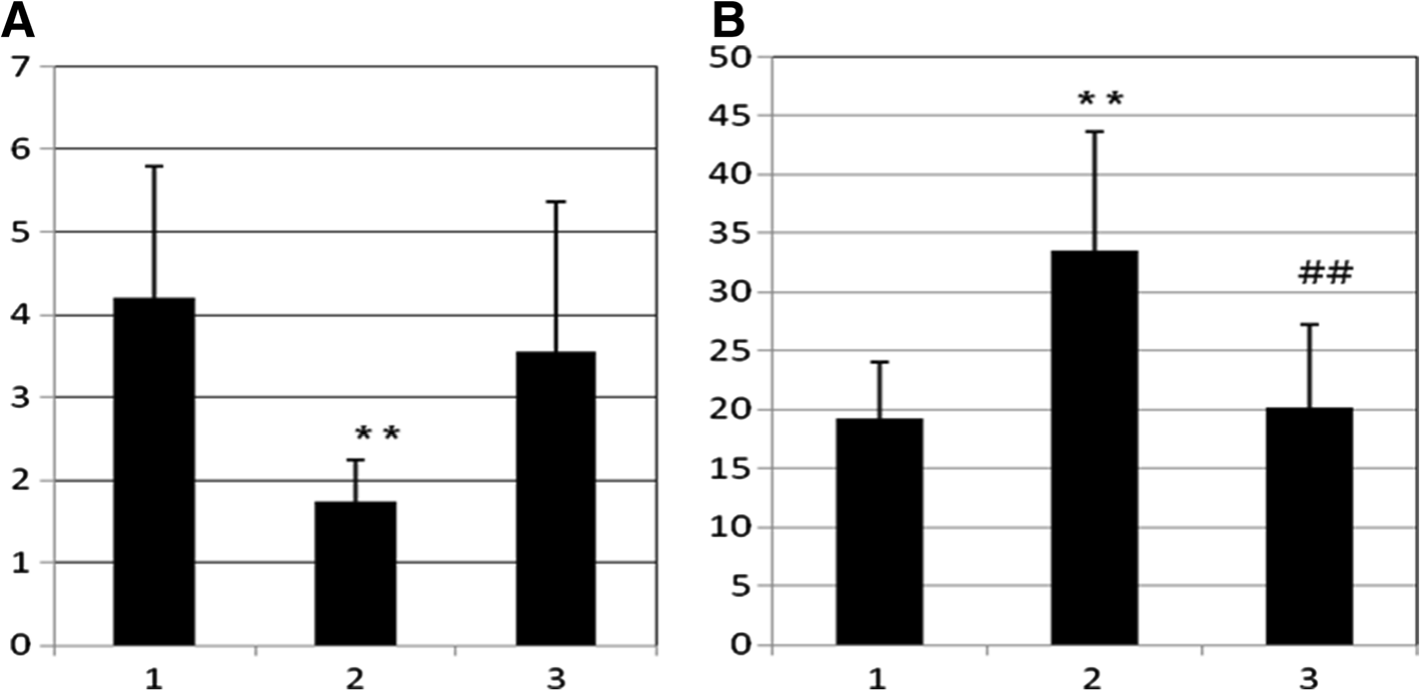
**Concentration of adipose-derived hormones. (A)** Concentration of adiponectin in serum (mkg/ml) and **(B)** concentration of leptin in adipose tissue of rats with glutamate-induced obesity and corrected by probiotics; double asterisks (**), *p* < 0.01 in comparison with group 1; double number signs (##), *p* < 0.01 in comparison with group 2.

The physiological function of leptin is to prevent obesity in excessive flow of food into the body. Reduced leptin secretion during fasting is a kind of signal to increase energy absorption. When there is excessive consumption of food, there will be increased activation of thermogenesis energy for the formation of brown fat by inducing the expression of genes responsible for the synthesis of mitochondrial proteins of type 1, 2 and 3; severe oxidative phosphorylation occurs, regulating the rate of thermogenesis in the body [38]. Analysis of the concentration of leptin in adipose tissue in rats that were administered in the neonatal monosodium glutamate showed an increase in this indicator by 74.7% (*p* < 0.01) compared to that of the intact animals (Figure 1). With obesity, serum leptin resistance increases due to the central hypothalamic action and lipocytokine mechanisms for negative feedback or defect transport through the blood–brain barrier. However, the effect of leptin on peripheral tissues preserved, so we can suspect the presence of selective leptin resistance. Tissues to leptin resistance develop gradually, activating the growth of adipose tissue [38]. It has been shown that the introduction of MSG causes lesions in the arches and ventromedial nuclei of the hypothalamus, causing insensitivity to leptin and insulin in this region resulting in developing hyperleptinemia and hyperinsulinemia [39].

Probiotic therapy performed to animals that received MSG (4 mg/g) at 2, 4, 6, 8 and 10 days of life prevented the development of obesity in rats. Thus, in rats injected with probiotic mixture, there is increased body length by 6.1% (*p* < 0.05) compared to that of the placebo and not different from that of the intact controls. Proof of reducing obesity index was significantly reduced Lee index and visceral fat mass.

Probiotic consumption also increased the adiponectin level, decreased the leptin concentration in adipose tissue and restored the anthropometric parameters and lipid metabolism (Tables 1 and 3; Figure 1). Such results are consistent with other studies and show effectiveness of probiotics in obesity prevention.

*Periodic administration* of probiotics led to restoration of lipid metabolism in rats. The probiotic strains have influenced the cholesterol concentration, which has been restored to the level of control. In organism of animals, injected with probiotic strains, the VLDL cholesterol concentration decreased by 32.3% (*p* < 0.05), LDL cholesterol by 30.6% (*p* < 0.05) and HDL cholesterol increased by 25.7% (*p* < 0.05) compared with those of the group 2. These values did not reach the level of control (Table 2). Probiotics introduction led to the normal hormonal activity of adipose tissue.

Thus, intermittent administration of probiotics, namely for 2 weeks, resulted in increased adiponectin levels, decreased leptin concentrations in adipose tissue and recovery of bodymetric parameters and lipid metabolism in animals that were injected with monosodium glutamate as neonates. *The results show the effectiveness of probiotic therapy to prevent obesity, consistent with other studies*.

### Study limitation

The survey was conducted on animals. We administered MSG subcutaneously (8 ml/g); however, considering that MSG is a food additive, the *per os* administration (with mother milk for newborn animals) would be most preferable for the relevant modeling. The study did not consider an immunology, genetic, cellular regulation mechanisms, obesity-related conditions, namely diabetes, gout, autoimmunity, and many factors of obesogenic environment and other issues that may impact to obesity development. Research itself did not suppose the predictive and personalized approaches.

## Discussion

As the current research did not touch regulation mechanisms of obesity and related conditions, namely diabetes, gout, autoimmunity and many factors of obesogenic environment and other issues that impact to obesity development, we consider to overview the series of important obesity-associated topics, the impact of probiotics to the obesogenic environment to crystallize the predictive and personalized approaches in the field. Many mechanisms of obesity are still unclear; new pathways are still awaited and insufficiently studied and should be extensively analyzed in conjoint paradigm. We hope that the discussion of the following issues for deeper understanding of certain mechanisms of obesity for effective translation results into real PPPM practice.

### Intestinal microbiota and obesity

The gut microbiota can be considered an extension of the self and, together with the genetic makeup, determines the physiology of an organism, metabolism and obesity [40]. Strachan [41] described the *hygiene hypothesis* that refers to an originally associated reduced microbial contact to microbes in early life and is suggested to be one of the main mechanisms to account for the increasing prevalence of allergic diseases over the past few decades. Today, reduced microbial exposures (and the rise in allergic conditions) have been attributed to Western lifestyle factors such as diet, antibiotic use, vaccinations, reduced household size and improved hygiene [42]. Recently, probiotic bacteria have been tested for their ability to affect obesity.

Previously, it was shown that the decrease of the cholesterol concentration in mice with high fat diet caused hypercholesterolemia under the influence of *Lactobacillus* та *Bifidobacterium*, especially *L. acidophilus ІМВ В-7279*, *B. animalis VKB* and *B. animalis VKL*[43].

The probiotic *Lactobacillus gasseri* SBT2055 was able to reduce adiposity and body weight in obese adults consuming a fermented milk with the bacterium for 12 weeks, potentially by reducing lipid absorption and inflammatory status [44]. A study by Luoto et al. [45] showed the effect of perinatal *Lactobacillus rhamnosus* GG on childhood growth patterns; the probiotic modulated the body weight increase in the early life but had no effect in later stages of development.

A recent study demonstrated that *Lactobacillus paracasei ssp paracasei F19* prevented diet-induced obesity in mice [46]. Lactoferrin (LF), a multifunctional glycoprotein in mammalian milk, is reported to exert a modulatory effect on lipid metabolism and improve visceral fat-type obesity, an underlying cause of the metabolic syndrome. Using a double-blind, placebo-controlled design, a study involved Japanese men and women (*n* = 26; aged 22 to 60 years) with abdominal obesity [47]. From these results, eLF appears to be a promising agent for the control of visceral fat accumulation with no adverse effects with regard to blood lipid or biochemical parameters. Therefore, influence of probiotics on obesity is actively debated, and we were the first who showed the preventive influence of the probiotic treatment in rat model with MSG-induced obesity.

However, the use of probiotics has several drawbacks; namely, introduction of foreign microorganisms induce antagonistic activity against pathogenic and indigenous microorganisms, and eliminating fast the probiotic strain. Therefore, to achieve the personalized approach, the development and application products manufactured from own strains of organism seem promising. For this reason, certain individual microorganisms might be grown on artificial nutrient, exploring their environment friendliness, establish spectrum antagonistic effects on the body. The potential alternative for probiotics might be the suggested lysates of probiotic strains that also exhibit immunomodulatory activity.

### Obesity genetics: monogenetic *vs* polygenetic concept

For over a decade, obesity genetics has been predominantly driven by research into *monogenic or syndromic obesity*.

Obesity tends to cluster within families and is more closely correlated between mono- and dizygotic twins. Studies of twins suggested that 80% of the risk of obesity is conveyed by genetic factors [48]. Some individuals are more predisposed than others to obesity-associated diseases, but it is presently difficult to identify the ‘at risk’ individuals who would benefit the most from individualized monitoring and care [49].

In the 1960s, Neel proposed the ‘thrifty gene’ hypothesis [50], whereby genes that predispose to obesity would have had a selective advantage in populations that frequently experienced starvation.

Recently, it was described a kinase suppressor of Ras 2 (ksr2), an intracellular scaffolding protein involved in multiple signaling pathways [49]. Targeted deletion of ksr2 leads to obesity in mice, suggesting a role in energy homeostasis as highly efficient in conserving energy. The expression of genes mediating oxidative phosphorylation is also downregulated in the adipose tissue of ksr2(−/−) mice [49]. This ‘hunger gene’ (ksr2) may cause continued hunger pangs in patients who are obese, as well as slow their metabolism. People who possess these genes in today’s obesogenic environment might be not just slightly overweight but becoming extremely obese [49]. These observations and our *in vitro* findings suggest that pharmacological approaches based on the modulation of KSR2 activity could represent a novel potential therapeutic strategy for the treatment of obesity and type 2 diabetes.

#### Rare familiar obesity

Rare familiar obesity include the *pure* forms of obesity, where the gene defect is in appetite regulation, and the disease is characterized by severe early onset obesity because of hyperphagia; syndromic forms have provided additional insights into the mechanisms underlying obesity [51,52]. Thus, cloning of the mouse ob gene and its human homologue, leptin [51], proved a paradigm of many genes involved in the regulation of appetite via the leptin-melanocortin pathway including leptin and its receptor, the α-melanocortin-stimulating hormone receptor (MC4R), pro-opiomelanocortin (POMC) and prohormone convertase-1 [52].

#### Polygenic obesity

The human obesity gene map [52,53] accumulated in the field of common polygenic obesity, 253 quantitative trait loci (QTLs) identified in 61 genome-wide scans and 52 genomic regions. There are currently 22 gene associations supported by positive studies [52-54]. These genes include members of the leptin-melanocortin pathway, pro-inflammatory cytokines and uncoupling proteins. The largest numbers of studies have been carried out on ADRB2 and PPARG, but the fact that they also have reported associations with asthma and T2D, respectively, may account for this [52]. The TCF7L2 association with diabetes demonstrate that sufficiently powerful studies can generate statistically strong results (*p* < 10^−8^) [54].

In both rare and common forms of obesity, that *epigenetic* influences, defined as any heritable influence on genes that occurs without a change in the DNA sequence, are also important, as the rare *Prader-Willi* and *Angelman* syndromes due to variations in genomic imprinting at the proximal long arm of chromosome 15 [52,55], gender-related differential responses related to obesity development [10].

### Panel of biomarkers for assessing obesity and associated disorders

It is promising to develop biomarkers, fed through different techniques, applicable to minimize the incidence of obesity and related disorders, and the morbidity and mortality it causes, leading to improve prevention and clinical management strategies. If well-characterized biomarkers were available, therapeutic intervention to prevent or delay the onset of obesity or its complications in susceptible individuals might become possible [56]. In addition, biomarkers might help us separate different sub-types of obesity, including those most associated with diabetes, cardiovascular disease and cancer. Some are prospective biomarkers for these different states of health and disease [57-60].

### Oxidative stress and obesity

Oxidative stress (OS) has been postulated as one of the main physiopathological hallmarks of most of chronic diseases, primarily neurodegenerative diseases, such as Alzheimer’s disease and other dementias, Parkinson’s disease, amyotrophic lateral sclerosis, epilepsy and multiple sclerosis [61,62], arthritis, cardiac dysfunctions and diseases that cause irreversible blindness, diabetic retinopathy, macular and retinal degeneration. Lastly, aging of any body is also associated with oxidative stress because the activity of the natural antioxidant system declines with age and the concentration of lipid peroxidation products increases.

Keaney and colleagues [63] report that smoking, diabetes and obesity are independently associated with increased oxidative stress in men and women in a large community-based cohort. Thus, the pro-inflammatory and pro-oxidant effects of increased adiposity represent a potential link between obesity and cardiovascular diseases.

There was an expressed idea that obesity is a state of chronic oxidative stress and inflammation. Oxidative stress induced by reactive oxygen species (ROS) is one of the main factors in cellular aging and many cellular disorders, which cause extensive damage to DNA [64]; also in mitochondria, it is an interesting promoting factor in virus-initiated carcinogenesis, metabolism and inflammation. In inflammation, ROS and nitric oxide (NO), generated by inflammatory cells, play a key role in carcinogenesis. Thus, ROS can induce the formation of 8-oxodG, an indicator of oxidative DNA damage, and NO, the formation of 8-nitroguanine, a marker of nitrative DNA damage. These factors are potentially mutagenic which may account for the cancer-promoting effect of inflammation. While therapeutic treatments cannot be based exclusively on the abatement of oxidative stress, neutralizing this cellular disorder could minimize collateral damages associated with the transformation of biomolecules in the cytosol.

#### Toxic visceral fat

Excess intra-abdominal adiposity has the potential to influence metabolism and cardiometabolic risk directly through alterations in the secretion of adipokines. Abdominal obesity promotes increased secretion of a range of metabolites and of biologically active substances, including glycerol, FFA, inflammatory mediators (e.g. tumor necrosis factor alpha (TNFα) and interleukin-6 (IL-6)), plasminogen activator inhibitor-1 (PAI-1) and C-reactive protein [65-67].

### Cardiovascular diseases

Obesity is well known to increase the risk of development of cardiovascular diseases. The evidence suggested an increase in the risk of ischemic heart disease associated with elevated plasma FFA (top vs lowest tertile) after correction for non-lipid risk factors, although further multivariate adjustment for lipid parameters and insulin weakened the association [67]. The majority of circulating FFA originates from upper-body subcutaneous adipocytes, whereas intra-abdominal fat content has been positively correlated with splanchnic FFA levels which may contribute to the liver fat accumulation commonly found in abdominal obesity [68,69].

*Adiponectin* has been shown to have many favorable metabolic properties [31-37]. The low adiponectin levels have been associated with adverse cardiovascular outcomes [65]. The secretion of adiponectin, an apparently cardioprotective adipokine, has been shown to be reduced in abdominally obese patients [68]. Leptin is an adipokine involved in the regulation of satiety and energy intake [38,39,65-69]; levels of leptin in the plasma increase during the development of obesity and decline during weight loss.

Cardiovascular diseases are strongly connected to immune response. Pathogenesis of cardiovascular diseases is associated with dysfunction of cytokine production. In most autoimmune diseases observed, stereotyped response in the form of a large subpopulation of activated Th1 lymphocytes [70], not rarely observed, decrease in the number of T lymphocytes, impaired T helpers/suppressors ratio downward suppressor activity, weakening the response to the mitogens. In patients with autoimmune disease, frequent increased levels of pro-inflammatory cytokines (TNF-α, IL-1, IFN-γ) may result in aberrant activation of the innate immune response [71]. During a persistent heart muscle damage, the exposure of the intracellular content to dead cells activates the innate immune response, such as the activation of Toll-like receptors (TLR). In the heart, TLR2 and TLR4 are perhaps involved in the host response to myocardial infarction [72]. The activation of TLR initiates the imbalance of TLR-induced cytokines regulation [73].

### Atherogenesis

Atherosclerosis is a chronic inflammation in the blood vessels, resulting in the buildup of fatty streaks, and with time, atherosclerotic plaques. Obesity, insulin resistance and high blood pressure are the risk factors for the disease. The gut microbiota has been suggested to play a significant role through its processing of phosphatidylcholine in the diet, leading to pro-atherogenic metabolites in [74]. Atherosclerosis has been shown to have an inflammatory component, and pro-inflammatory adipokines may be important mediators of atherogenesis in abdominally obese subjects [65]. The anti-atherogenic actions of adiponectin appears to be multifactorial, including inhibition of endothelial activation, reduced conversion of macrophages to foam cells and inhibition of the smooth muscle proliferation and arterial remodelling that characterizes the development of the mature atherosclerotic plaque [75].

### Diabetes, insulin resistance

Steppan et al. described links between obesity and diabetes by hormone named resistin (for resistance to insulin), a unique signaling molecule, adipocytes secrete [76]. It was demonstrated the improvement of lipid metabolism in the case of probiotic consumption [77]. For example, the modulation of gut microbiota, e.g. dietary intervention with oligofructoses, reduced metabolic endotoxemia and the cecal content of LPS, improved glucose intolerance, insulin sensitivity and decreased body weight gain in both high-fat fed and ob/ob mice [78].

In models of diabetes, probiotic intervention has been examined for its ability to impact on metabolic biomarkers of disease. Tabuchi et al. showed that *Lactobacillus rhamnosus* GG improved glucose tolerance in the streptozotocin-induced rat model of diabetes possibly due to the prevention of insulin secretion decrease [79]. Studies using the traditional Indian yoghurt, dahi, supplemented with probiotic strains of Lactobacillus acidophilus and L. casei have shown that this product can improve markers of diabetes, including hyperglycemia, and hyperinsulinemia in high-fructose-induced rat models of diabetes [80,81].

For instance, it improves insulin sensitivity and glycemic control [82], and levels of this adipokine correlate positively with levels of HDL cholesterol and inversely with TG or PAI-1 [83].

Type 1 diabetes (T1D) is considered as a debilitating autoimmune disease that results from T cell-mediated destruction of insulin-producing beta cells. The innate immune system is another factor that probably influences the composition of the intestinal microbiota. This is best illustrated by a study showing that diabetic mice deficient in the innate signaling molecule MyD88 are protected from the development of type 1 diabetes [65,84].

### Inflammation

Inflammation is a key feature of obesity and type 2 diabetes [85], being a source of oxidative stress, which is also implicated in the development of atherosclerosis. Elevated levels of plasma and urinary F2-isoprostanes have been found in a number of inflammatory diseases [65,86]; increased production of reactive oxygen species may also enhance the inflammatory response by activating redox-sensitive nuclear transcription factors such as AP-1 and NF-κB. These transcription factors are essential for the inducible expression of genes associated with immune and inflammatory responses, including cytokines, cell adhesion molecules and inducible NO synthase. Thus, the pro-inflammatory and pro-oxidant effects of increased adiposity represent a potential link between obesity and CVD. F_2_-isoprostanes are prostaglandin-like products of the free radical-catalyzed peroxidation of arachidonic acid. They are formed *in situ* esterified to phospholipids and are released into plasma by phospholipases [87].

*The non-steroidal anti-inflammatory drugs as* diclofenac are associated with the formation of reactive metabolites and hepatotoxicity [88]; the potential risks of non-steroidal anti-inflammatory drug for obesity development is promising but not yet studied.

### Atopic diseases and obesity

Most of the studies point out that obesity is capable of increasing the prevalence and incidence of asthma, although this effect appears to be modest. The treatment of obese asthmatics must include a weight control program [89].

Obesity is capable of reducing pulmonary compliance, lung volumes, and the diameter of peripheral respiratory airways as well as affecting the volume of blood in the lungs and the ventilation-perfusion relationship. Furthermore, the increase in the normal functioning of adipose tissue in obese subjects leads to a systemic pro-inflammatory state, which causes a rise in the serum concentrations of several cytokines, the soluble fractions of their receptors and chemokines. Many of these mediators are synthesized and secreted by cells from adipose tissue and receive the generic name of adipokines, including IL-6, IL-10, eotaxin, tumor necrosis factor-alpha, transforming growth factors-beta1, C-reactive protein, leptin and adiponectin.

According to the hygiene hypothesis, it asserts that the increase in the prevalence of atopic disease is related to reduced exposure to microbes at an early life [40-42].

According to the study by Shinkai et al. [90], type-2 immunity requires orchestration of innate and adaptive immune responses to protect mucosal sites from pathogens. Dysregulated type-2 responses result in allergy or asthma. T helper 2 (T(H)2) cells elaborate cytokines, such as IL-4, IL-5, IL-9 and IL-13, which work with toxic mediators of innate immune cells to establish environments that are inhospitable to helminth or arthropod invaders.

The importance of T(H)2 cells in coordinating innate immune cells at sites of inflammation is not known. Here, we show that polarized type-2 immune responses are initiated independently of adaptive immunity. In the absence of B and T cells, IL-4-expressing eosinophils were recruited to tissues of mice infected with the helminth *Nippostrongylus brasiliensis*, but eosinophils failed to degranulate. Reconstitution with CD4 T cells promoted accumulation of degranulated IL-4-expressing cells, but only if T cells were stimulated with cognate antigen. Degranulation correlated with tissue destruction, which was attenuated if eosinophils were depleted. Helper T cells confer antigen specificity on eosinophil cytotoxicity, but not cytokine responses, so defining a novel mechanism that focuses tissue injury at sites of immune challenge [40,73,89-91].

The cytokine interleukin-17 (IL-17) has received considerable attention since the discovery of a distinct CD4(+) T helper (T(H)) cell subset that produces it, known as the T(H)17 cell subset. Despite the fact that most of the recent literature describes IL-17 as a T cell-secreted cytokine, much of the IL-17 released during an inflammatory response is produced by innate immune cells. In this review, we explore the many innate immune cell populations that are an early source of IL-17 in response to stress, injury or pathogens. These early sources have been shown to have a central role in the initiation of IL-17-dependent immune responses, even before the first CD4(+)T cell sees its cognate antigen and initiates the T(H)17 cell developmental program [90,91].

Both systemic and myeloid tissue-specific A(2B) R deletion significantly decreased pulmonary inflammatory cell recruitment, airway mucin production and pro-inflammatory cytokine secretion after final allergen challenge in sensitized mice. A(2B) R deficiency resulted in a dramatic reduction on Th2-type airway responses with decreased pulmonary eosinophilia without augmenting neutrophilia and decreased lung IL-4, IL-5 and IL-13 production. Pro-inflammatory TNF-α, IFN-γ and IL-17 secretion were also reduced in systemic and myeloid tissue-specific A(2B) R deletion mouse lines [92]. This Th2-type dysfunction in bronchial asthma inclines to use immunocorrection that can be achieved with probiotics based on Gram-positive microorganisms [93,94].

### Infection-related metabolic conditions

It was speculated that adenovirus 36 (Adv36) not only infects humans but also can make them obese [95]; studies in humans and experiments in animals supported this hypothesis. There were illustrated extensive interrelations among virus action, cellular oxidative stress, gene damage, multiple immune pathways and proteomic changes in diabetes mellitus, cancer and many chronic disorders development; many of them are also related to HPV infection [96,97]. On the other hand, the obesity may induce the T cell response in virus infection [98]. Patients with increased BMI and adiposity also present a higher incidence of surgical site infections with increased risk of wound complications [85,99].

### Gender and obesity

Findings showing women’s differential metabolic responses have suggested a gender effect on biochemical-endocrinological patterns, metabolic mechanisms and risk factors, emphasizing the importance of more gender-specific prevention strategies [10]. This is especially relevant vs environmental changes and the obesogenic epidemic, with women’s lead in earlier and higher obesity rates and related disease risk, though with manifestation mostly delayed to menopausal age. Estrogen receptors (ER) are well-known regulators of several aspects of metabolism, including glucose and lipid metabolism, and impaired estrogen signaling is associated with the development of metabolic diseases [10,100]. Here, ERα seems to play a protective role in insulin and glucose metabolism through effects on the liver, adipose tissue, muscle, and pancreatic β cells and on central regulation of food intake and energy expenditures. ERβ, on the other hand, has the potential to negatively influence insulin and glucose metabolism by impairment of adipose tissue function. The onset of menopause dramatically increases the risk for women to develop disease states coupled to the metabolic syndrome, such as obesity, CVD, and type 2 diabetes [10]. The endometrial receptor status being strongly associated with metabolic mechanisms contribute the diagnostic algorithm as potential biomarkers for pathogenetic therapy. Thus, in determining relationships in the endometrial receptor system, each individual pathological pattern of receptors and their relationship further defines personalized pathogenetic tactics tailored to the person [100].

### Hyperuricemia, gout

Hyperuricemia, which is strongly associated with obesity and metabolic syndrome and can predict visceral obesity and insulin resistance, might be partially responsible for the pro-inflammatory endocrine imbalance in the adipose tissue, which is an underlying mechanism of the low-grade inflammation and insulin resistance in subjects with the metabolic syndrome [101] and is discussed to be involved in the mechanism of MSG metabolism. Gutman in 1973 [102] expressed the hypothesis that, possibly secondary to altered glutamate catabolism, glutamine apparently is overutilized for urate overproduction by the liver and underutilized for reduced renal ammonia formation. The kidneys, modulating urinary uric acid excretion, sustain the hyperuricemia. In these considerations of the possible role of glutamine, another factor should now be taken into account, namely, the hyperglutamatemia of primary gout. Pagliara and Goodman suggested that in gout, the impaired catabolism is due to a partial deficiency of glutamate dehydrogenase; gouty patients had raised levels of plasma glutamate [103]. Certain foods and food additives are used along with monosodium glutamate, or MSG, to enhance the flavor of bland food, and contain purines, which are directly metabolized into uric acid, as guanylate (E626, E627, E628 and E629), inosinate (E630, E631, E632 and E633), and their compounds ribonucltides (E634 and E635) are metabolized to purines. More studies are required to establish the links with food additives and crystal deposition diseases, prior to gout.

Gout is characterized by the deposition of reversible *monosodium urate* (MSU) crystals and occurs as a consequence of hyperuricemia [104] which induces inflammatory arthritis [105] and nephropathy [106] and has risen over the last decade. Dr. Veronique Vitart, from the Medical Research Council Human Genetics Unit at the University of Edinburgh, researcher of hyperuricemia genetics said: ‘*Abnormal levels of uric acid have been associated with various common diseases and conditions, but causal relationships are not always clear. Gaining insight into the genetic components of uric acid levels offers a very useful tool to tackle these issues and to further our understanding of these conditions.*’ In the study by Köttgen et al., including 110,347 individuals from 48 studies were suggested and 28 genome-wide significant loci associated with serum urate concentrations were identified. Those alleles associated with increased serum urate concentrations were also associated with increased risk of gout. The modulation of urate production and excretion by signaling processes that influence metabolic pathways, such as glycolysis and the pentose phosphate pathway, seems to be central pathways including the genes from the newly identified associated loci. These findings may have implications for further research into urate concentration, lowering drugs to treat and prevent the common inflammatory arthritis gout [107].

Advanced imaging modalities including MRI, ultrasound (US), CT and dual energy CT have important applications in gout. US and MRI also reveal the severity of inflammation within and adjacent to the joint and can capture information about the composite, vascular nature of many tophaceous deposits [104,108]. According to our recent data [109], ultrasound can be an effective method for early detection of liver and kidney involvement in gout patients with sensitivity, specificity, positive and negative predictive value and accuracy; the gout involvement of liver and kidneys using complex ultrasonography diagnostic criteria were 92.6%, 84.4%, 80%, 95% and 91.9% respectively. We suggested the integrated index calculated by suggested mathematical model according to which the process (disease progression) is described by the primary indicators (biomarkers) that with output rate are stochastic in nature and presented as statistical information. A special algorithm for processing statistical data could be reliable for disease staging and control treatment follow up. We recommended to create the system for complex evaluation of biomarkers using suggested mathematical model based from patient medical records for prediction, personalized treatment and prevention of gout in relation to metabolism and obesity that may be applicable in still existing closed medical system, obtaining relevant extensive data.

### Immune pathways

The ability of *probiotics*, which affects the relevant Toll-like receptors (TLRs), can promote effective immune response and the initiation of an effective immune defense [73]. Gram-positive bacteria affect the formation of T and B cell immune response by altering products primarily; IFN-γ and IL-12 are required for differentiation of T helper cells into Th1 subpopulation direction. However, probiotic preparations are capable of activating both (Th1 and Th2) lymphocyte subpopulations, which provide a balance of cytokine production. Immunomodulatory activity of probiotic preparations is most important to identify the goods which induced opposite cytokines IL-10 or IL-12 in experiments *in vitro* when stimulating macrophage cells. The immune response against infectious diseases of probiotic drugs is activated due to the ability to balance the body’s immune status at the level of receptor-ligand interactions. Probiotic preparations are the agonists TLR-2, and the presence of common protein adapter molecules (TIRAP), MyD88 for TLR-2 and TLR-4 to influence the signaling pathways of cytokine production under the influence of the ligands. Inhibition of TLR2-induced signaling, influenced by lactobacilli via adapter Mal/MyD88, can lead to partial inhibition of TLR4 signal, accompanied by decreased production of pro-inflammatory cytokines. Ability to influence signaling pathways of cytokine production opens new perspectives for the creation of probiotic preparations with anti-inflammatory properties.

According to our recent data [110], daily oral administration of strains *L. rhamnosus V*® or *L. rhamnosus* LB3 IMB B-7038 (1 × 10^6^ cells / mouse) for 4 days and infected *Staphyloccous aureus* 8325 mice in a dose (5 × 10^8^ CFU/mouse) LD50 was accompanied by a reduction in the mortality of animals. The introduction of lactic acid bacteria to the animals infected with *S. aureus* allowed increased functional activity of phagocytic system and the normalization parameters of cellular immunity activation and production of interferon-γ and IL-12 in different periods of observation. However, decreased production of IL-4 indicates the ability of probiotic strains to balance the immune response in the upward cytokine production by Th1-type, which guide the development of the immune response to cell type. The introduction of lactic acid bacteria accompanied the strengthening of the ability of splenocytes to produce IFN-α and IFN-γ in response to adequate stimulation. Strengthening biocide activity of macrophages and cytotoxicity natural killer under the influence of experimental strains leads to a significant increase of elimination *S. aureus*-infected kidneys of animals.

These data suggest that lactic acid bacteria may dynamically modulate the mechanisms of innate and adaptive immunity by maintaining the balance between Th1 and Th2 lymphocytes, which allows us to consider them as immunomodulatory drug selective action [108].

### Associations with neurodegenerative, musculoskeletal diseases and pain

Interferon-b is an established treatment for patients with multiple sclerosis (MS), but its mechanisms of action are not well understood [111]. Viral infections are a known trigger of MS relapses. Toll-like receptors (TLRs) are key components of the innate immune system, which sense conserved structures of viruses and other pathogens [73]. The upregulation of TLR7 in pDCs and a consequently increased activation of pDCs by TLR7 ligands represents a novel immunoregulatory mechanism of interferon-b. Derkow et al. hypothesized that this mechanism could contribute to a reduction of virus-triggered relapses in patients with MS [111].

Many studies clearly suggest that TLRs, acting through MyD88-dependent and/or independent mechanisms, induce pro-inflammatory signals for the development of myopathy in skeletal muscles. Thus, the emerging paradigm indicates that not only innate and adaptive immune mechanisms but also intrinsic defects in myopathic skeletal muscle contribute to muscle weakness and damage such as myositis. The muscle microenvironment is complex, consisting of active interactions occur between innate, adaptive, metabolic and homeostatic pathways in the muscle in these diseases [112].

Acute exposure of skeletal muscle to elevated levels of FFA induces insulin resistance, whereas chronic exposure of the pancreas to elevated FFA impairs β-cell function [113,114]. Obesity-related postural mechanism, neuromuscular networks and pain muscle and immune-neurodegenerative relationships are still unclear and subject for further studies [115].

### Vasospasm, congestion and obesity

Endothelial dysfunction (ED) induced by obesity is an important risk factor that impairs blood flow controls in various organs. Obesity impairs microvascular function in several ways. ED results from an imbalance between NO and endothelin (EDN), being the regulators of vascular function. Obesity-induced ED is associated with decreased NO production due to impaired endothelial NO synthase activity and expression and increased production of superoxide anion and the endogenous NOS inhibitor ADMA, together with increased vasoconstrictor factors, such as endothelin-1 and sympathetic nerve activation [116].

Genetic variants in NO synthase and EDN isoforms and its receptors (EDNRA and EDNRB) appear to account for important components of the variance in ED, particularly when concurrent risk factors such as obesity exist. Analysis of genotype-phenotype interactions is critical for the formulation of the potentially altering predisposition to cardiovascular diseases [117]. NO synthase and endothelin genes are related with many diseases, namely, asthma [118] and renal failure [119], that make them the potential biomarkers of numeral obesity collateral pathologies.

Meyer et al. found that in the aorta of obese mice, perivascular adipose potentiates vascular contractility to serotonin and phenylephrine, indicating the activity of a factor generated by perivascular adipose, which we designated as ‘adipose-derived contracting factor’ (ADCF) [120]. Inhibition of cyclooxygenase (COX) fully prevented the ADCF-mediated contractions, whereas COX-1 or COX-2-selective inhibition was only partially effective. By contrast, inhibition of superoxide anions, NO synthase, or endothelin receptors had no effect on ADCF activity [120].

Congestive mesenteric [121] and/or pelvic syndromes (ovarian vein reflux) [122] are the condition characterized by the presence of venous congestion and varicose veins in the mesenteric and pelvic region and play important role for dysregulation of intestinal and systemic microcirculation mechanisms leading to ED in overweight patients and have potential risk for the development of many vascular and hormonal disorders related to obesity. In obese individuals, the mixed meal drink decreases the baseline skin perfusion and causes acetylcholine-mediated vasodilation but has no effect on the capillary density. Obese individuals had impaired acetylcholine-mediated vasodilation after meal ingestion. The latter findings are consistent with impaired postprandial microvascular function in obesity [123].

Peripheral microcirculation assessment might be considered to support a supplementary information for obese patients particularly for vasospasm assessment [124], including laboratory biomarkers and capillaroscopy [125]; Doppler techniques for assessment of vascular responses following cuff-induced arterial occlusion allow determinations of the kinetics of post-ischemic reperfusion and provides an accurate reporter of NO-mediated physiological recruitment [117].

### Nanotechnologies - the challenge for advanced diagnosis, treatment and prevention

Advances in nanoscience, nanotechnology and nanomedicine lead to the construction of new materials and devices for various scientific and therapeutic purposes which are applicable in molecular diagnostics, nanodiagnostics and improvements in the discovery, design and delivery of drugs, including nanopharmaceuticals. The application of nanoparticles allowing the combination of therapy and diagnosis, known as theranostic, has received increasing attention in biomedicine [126]. Pharmacological, pharmaceutical and toxicological aspects of the application of nanoparticles in biomedical purposes still remain poorly understood. While oxidative stress has been postulated as one of the main physiopathological hallmarks of most of chronic diseases, the nanoparticles of gold [126,127] and cerium dioxide [118,119] were reported as strong agents against oxidative damage having anti-aging activity. Nanoparticles of cerium dioxide, considering its UV-shielding effect, antiviral, antibacterial, antifungal activity, cardioprotective, neurotrophic, hepato- and nephroprotective and anti-aging effect, have potential for various biomedical applications [126-129]. Treatment with nanoceria has supplementary perspectives in gynecology and reproductive medicine and also in women with hormone-associated obesity which results from the increase in the number of oocytes in follicles, increase in the number of oocytes at metaphase I and metaphase II, increase in the number of living granulosa cells and decrease in the number of necrotic and apoptotic cells [128,129].

### Potential economical impacts

Obesity as a condition associated to many pathologies accounts for the burden related to the treatment of these preventable diseases (about €59 billion a year in EU; US$71.1 billion in the USA). The combined medical costs attributable to obesity and overweight are projected to double every decade and will account for 16% to 18% of the total US health care expenditure by 2030 [130]. Thus, considering integrative medical approach against obesity within the PPPM paradigm should lead to significant *economic benefits*.

### Education for preventive measures

Educational programs and individual preventive facilities are the important tasks for the PPPM concept in metabolic disorder early *risk factors detection and early obesity environment programming*. A number of potential effective plans can be implemented to target built environment, physical activity and diet. Primary or secondary prevention of obesity and these strategies are more effective in children than in adults [131]. These strategies can be initiated at home and in preschool institutions, schools or after-school care services as natural setting for influencing the diet and physical activity and at home and work for adults. Dissemination of information is necessary in order to popularize dietary recommendations, screening programs and patient participation approaches against risk factors including obesity, fat diet, diabetes, women health and family history. The material for dissemination and lecturing should be culture specific and ethnically standardized to the socio-economical aspects of the targeting population (well translated, to be easily understood) in order to facilitate the work. Development and implementation of linguistically validated evidence-supported questionnaires are required to assessment conditions specific to obesity which is tailored to the person. Support of preventive educational activity with long-term commitment of private and public funding programs is required.

### Consolidation of the PPPM concept

#### Personalized medical approach

Avoiding adverse additive and designing person-related probiotic strains are important impacts to personalized dietology.

#### Predictive medical approach

Translating obtained data on animal model to human organism may allow to consider consumption of the products containing MSG, especially in early age, as a predictive value for obesity and metabolic syndrome development. Developing the panel of obesity assessment biomarkers is an important point.

#### Preventive medical approach

Translation of the obtained data on animal model to human organism may allow to consider diet correction with probiotics within early obesity environment programming as a strong preventive measure against obesity epidemic.

## Expert recommendations

### Conclusion

The introduction of monosodium glutamate to newborn rats causes obesity in adulthood. Periodic administration of probiotic blends to rats that received MSG in neonatal period prevented the development of obesity.

### Future outlooks and recommendations

Further studies including sufficient cohorts of volunteers are necessary to translate model data of probiotic efficacy to human population as well as conduction of epidemiological studies for obtaining evidence regarding adverse effect of particular food additives.

We also suggest the further studies regarding obesity and metabolic disorders inducible by expression of genes associated with obesity environment, dietary additives, immune and inflammatory responses, and develop and test promising nanomaterials (e.g. nanoceria) for prospect of complex impact to gastrointestinal microbiota and motility.

Create the relevant models to study microbiota related to specific process (diabetes, gout, hepatitis, cancer, CVD, etc.). Obtained results may indicate the effectiveness of probiotic therapy to prevent obesity in human based on personalized microbiota additives. To achieve the personalized approach, the development and application products manufactured from own strains of organism are considered promising. The potential alternative for probiotics might be the suggested lysates of probiotic strains that also exhibit immunomodulatory activity.

After approval, development of safe and effective person-related medications/additives with future clinical testing should be initiated to implement results for routine practice.

With the concluding points, we can formulate the following proposals (expert recommendations):

1. For the European Union (EU): create an international project to study the effects of probiotics for the implementation of evidence-based personalized dietology against obesity and metabolic syndrome.

2. For Ukraine: participate in the project in partnership with EU to follow up experimental and clinical trials and involve related institutions and centres to the study.

## Abbreviations

CFU: colony-forming units; CVD: cardiovascular diseases; FFA: free fatty acids; HDL cholesterol: high-density lipoprotein cholesterol; LDL: low-density lipoprotein; MSG: monosodium glutamate; PPPM: predictive preventive and personalized medicine; VLDL: very low-density lipoproteins; WHO: World Health Organization.

## Competing interests

The authors declare that they have no competing interests.

## Authors' contributions

SOA, VOV and FTM performed experiments and statistical analysis of obtained data and prepared the article. BTV did the organization, literature review and analysis of the study. LLM performed experiments and analysis of the study and prepared the article. RVB participated in the analysis of the study, did the literature review in part of the discussion, formulated prospects and performed the final article drafting. MYS did the organization and analysis of the study and prepared the article. All authors read and approved the final manuscript.

## Authors' information

SOA, VOV, FTM, Ph.D., D.Sci., and Professor BTV, Ph.D., D.Sci. are researchers of SRL ‘Pharmacology and Experimental Pathology’, Department of Biological and Biomedical Technology, ESC ‘Institute of Biology’, Taras Shevchenko National University of Kyiv. Professor LLM, Ph.D., D.Sci. is a researcher in Inteferon Department of Zabolotny Institute of Microbiology and Virology, National Academy of Sciences of Ukraine. RVB, M.D., Ph.D. is a medical doctor in the Clinical Hospital ‘Pheophania’ of the State Affairs Department, researcher of the Inteferon Department of Zabolotny Institute of Microbiology and Virology, National Academy of Sciences of Ukraine, and National Representative of the European Association for Predictive, Preventive and Personalised Medicine (EPMA) in Ukraine. Professor MYS, Ph.D., D.Sci. is a corresponding member of the National Academy of Sciences of Ukraine and the director of the Inteferon Department of Zabolotny Institute of Microbiology and Virology, NAS of Ukraine, Kyiv, Ukraine.
